# Hunting for Toxic Industrial Chemicals: Real-Time Detection of Carbon Disulfide Traces by Means of Ion Mobility Spectrometry

**DOI:** 10.3390/toxics8040121

**Published:** 2020-12-14

**Authors:** Victor Bocos-Bintintan, Ileana Andreea Ratiu

**Affiliations:** 1 Faculty of Environmental Science and Engineering, Babes-Bolyai University, RO-400294 Cluj-Napoca, Romania; 2 “Raluca Ripan” Institute for Research in Chemistry, Babes-Bolyai University, RO-400294 Cluj-Napoca, Romania; 3 Interdisciplinary Centre of Modern Technologies, Nicolaus Copernicus University, 87-100 Toruń, Poland

**Keywords:** carbon disulfide, ion mobility spectrometry (IMS), photoionization detection (PID), toxic industrial compounds (TICs)

## Abstract

Sensitive real-time detection of vapors produced by toxic industrial chemicals (TICs) represents a stringent priority nowadays. Carbon disulfide (CS_2_) is such a chemical, being widely used in manufacturing synthetic textile fibers and as a solvent. CS_2_ is simultaneously a very reactive, highly flammable, irritant, corrosive, and highly toxic compound, affecting the central nervous system, cardiovascular system, eyes, kidneys, liver, skin, and reproductive system. This study was directed towards quick detection and quantification of CS_2_ in air, using time-of-flight ion mobility spectrometry (IMS); photoionization detection (PID) was also used as confirmatory technique. Results obtained indicated that IMS can detect CS_2_ at trace levels in air. The ion mobility spectrometric response was in the negative ion mode and presented one product ion, at a reduced ion mobility (K_0_) of 2.25 cm^2^ V^−1^ s^−1^. Our study demonstrated that by using a portable, commercial IMS system (model Mini IMS, I.U.T. GmbH Berlin Germany) one can easily measure CS_2_ at concentrations of 0.1 ppm_v_ (0.3 mg m^−3^) in the negative ion mode, which is below the lowest threshold value of 1 ppm_v_ given for industrial hygiene. A limit of detection (LOD) of ca. 30 ppb_v_ (0.1 mg m^−3^) was also estimated.

## 1. Introduction

Ion mobility spectrometry (IMS) is a rapid, highly performant, and unique analytical technique, which is widely used for separating and identifying chemicals as vapors present in air samples, at trace and ultra-trace levels, after these chemicals are ionized at atmospheric pressure [1,2]. The IMS technique has an exceptional sensitivity, since it measures ionic currents in the pico-amperes (10^−12^ A) range [1,2,3,4]. The rapidity of ion separation in the gaseous phase and at atmospheric pressure is the principal advantage of ion mobility spectrometry (a single spectrum is being acquired in just several dozens of milliseconds), which combines perfectly with the outstanding sensitivity (in the low ppb_v_ range for most chemicals). Moreover, the great comfort in the use of IMS instrumentation, which has also a compact, rugged, and miniaturized design, determines the increasing popularity of IMS—from sensing explosives [5,6], precursors of illegal drugs [7,8], or chemical weapons [9,10], to an increasing number of bio-medical [11,12] and industrial applications [13,14]. Moreover, the IMS technique has brought benefits in various fields, such as medicine, biology, industrial hygiene, security applications, and investigation of the environment [6].

Carbon disulfide is a linear non-polar molecule, a colorless liquid (with a pleasant smell—sweet—if it is pure; reagent grades are foul smelling) or faintly yellow (with an unpleasant odor like that of rotting radishes when it is impure), with moderate solubility in water and high lipophilicity [15,16]. The most important chemical and physical properties of CS_2_ are listed in Table 1.

Although CS_2_ is well known as a natural component released from geological oil and gas deposits, is naturally produced by microorganisms in the soil, or emitted as a result of vegetation fires and volcanoes [17,18]; however, the main sources of CS_2_ are anthropogenic. Therefore, CS_2_ is a toxic industrial compound (TIC), produced by the heating/burning of all carbon sources containing sulfur (charcoal, natural gas, oil) [15] and widely utilized in several important industries. For instance, the annual industrial production of CS_2_ was evaluated in 2010 to approximatively 75 million kilograms per year, only for use in rayon and cellophane production, as well as in oil, gas, agricultural, and metal processing industries [17]. 

The toxicity of CS_2_ has been described since 1850, by the French medical doctor August Delpech, who described that the abovementioned toxin causes the so-called “disulfide carbon neurosis” [19]. Later on it was revealed that the most relevant health problems connected with CS_2_ exposure are those related to the cardiovascular system and central nervous systems, as well as to retinal angiopathies and impairments of color vision [20]. Toxic effects on the female reproductive system, (menstrual disturbances and precocious menopause occurrence) that appeared in workers chronically exposed to CS_2_ in their workplace in the viscose industry were also well documented and discussed in a comprehensive review article [21]. An epidemiological study that extended over 10 years (1975–1985), including 251 workers exposed to CS_2_ and 124 controls, was realized in two viscose rayon factories in Czechoslovakia. The obtained results highlighted the increased mortality due to cardiovascular diseases of spinner workers exposed to high levels (between 9.6 and 48 ppm) of CS_2_ when compared with the less exposed workers [22]. Today, the neurotoxicity of CS_2_ [23], its vascular effects [24], cardiovascular and liver effects [25], and other effects [22] are well documented. The effect of prolonged exposure to CS_2_ in humans is summarized in Table S1, which is presented as Supplementary Material.

The OSHA permissible exposure limit (8-h time-weighted average (TWA)) is 20 ppm_v_ (ca. 73 mg CS_2_ m^−3^ of air), while the recommended exposure limit (REL) for REL-TWA (time-weighted average) is only 1 ppm_v_ (3.16 mg m^−3^); REL-STEL (short-term exposure limit) is 10 ppm_v_ (31.6 mg m^−3^). The NIOSH recommendations are as the follows: the recommended exposure limit: 1 ppm_v_ (3.16 mg m^−3^)—skin (10 h TWA); recommended exposure limit as 15 min short-term exposure limit (STEL): 10 ppm_v_ (31.6 mg m^−3^)—skin. The oral lethal dose for a human was reported to be as low as 15 mL for an adult (upon ingestion of CS_2_), so the LD_50_ = ca. 250 mg CS_2_/kg body weight. The minimum lethal dose reported in humans was only 14 mg/kg body weight (1 g of CS_2_ for a 70 kg person). However, the exposure to 500 ppm_v_ (for 30 min) can be life-threatening (IDLH—Immediately Dangerous to Life and Health; NIOSH) [26,27]. Death has been reported from exposure to a CS_2_ vapor concentration of 4815 ppm for 30 min [28]. 

Persistence of CS_2_ in the environment is rather low. The life time of CS_2_ in the air roughly ranges from 1 to 10 weeks [22]. The average decomposition half-time (t_1/2_) of CS_2_ vapors in the atmosphere is about 5 to 10 days [28]. The aquatic fate of CS_2_ does not imply its adsorption onto sediments or suspended solids, but rapid volatilization with half-lives ranging between 3 h and 2.5 days, a function of concrete conditions [28]. The database on occupational exposure to CS_2_ as a result of rayon fiber production is extensive. In Table 2, some selected examples from studies conducted after the 1990s, reported in different countries, are indicated. In the last years, more and more care has been taken to reduce CS_2_ pollution in the environment, and consequently human exposure to it. The global problem is a significant one, and pollution control standards are increasingly restrictive [18]. To address this, fast and sensitive analytical platforms able to detect CS_2_ at trace levels (and preferably at lower concentrations than the dangerous thresholds) are highly required. GC-MS and GC-FID, equipped with various types of columns [29], which are considered the gold standard in VOC analysis used in various fields [30,31,32,33,34], have been involved in CS_2_ detection in human breath and proved to have a detection sensitivity of 76 ng m^−3^ in case of GC-MS [35] and 5 μg m^−3^ in case of GC-FID [36]. However, GC-FID methods are not the most suitable to analyze CS_2_ because it lacks C–H bonds, which are necessary for the production of a good signal in FID [37]. Classical spectrophotometric methods are also useful (with limits of detection of 0.2 and 0.4 mg⋅m^−3^, respectively [38]), but they involve a laborious and time-consuming sample preparation. Recently, an innovative method for analysis of CS_2_ using a Polyarc/FID system was developed; a minimum detectable concentration of 0.14 ppm_v_ was found [37]. 

We demonstrated through this study that IMS instrumentation is perfectly fitted for this type of application, presenting some strategic advantages (sensitivity, sensibility, and real-time response) compared with other techniques used before. Unspecific PID (photoionization detector) instrumentation, previously used for toxic VOCs monitoring in shoe shops [49] or soil contamination control [50], is also a suitable and useful technique to be used for quantification of CS_2_. The limits of detection of both PID and IMS are lower than the limits detected by the classically used instrumentation, while sample preparation is not required. The relevance of this manuscript relies on the importance of CS_2_ and to the related deleterious effects—on humans and on the environment—produced by its vapors. IMS has already proven to be a very useful and fast analytical technique for rapid trace detection of chemicals. We have proven that IMS can be used successfully to measure, in real time (several seconds), trace levels (sub parts-per-million, ppm_v_) of CS_2_ vapors in air. The minimum detectable concentration of CS_2_ was found to be around 100 ppb_v_ (0.31 mg/m^3^), which is well below the lowest threshold limit established for industrial hygiene, which is 1 ppm_v_. 

To the best of our knowledge, until now the detection and quantification of carbon disulfide using the IMS technique has not yet been described, and this is why the present work represents an original approach.

## 2. Materials and Methods 

### 2.1. IMS Instrument Used

A portable ToF (Time-of-Flight) IMS instrument (size: 26.5 × 22 × 14 cm; weighing 3.8 kg), providing real-time responses (ca. 1 s), model Mini-IMS, manufactured by the company I.U.T. mbH Berlin, Germany, was used for CS_2_ detection and quantification. The IMS cell has a classic design with stacked rings, where the conducting rings are alternated with insulating rings. The drift length of the IMS cell was 55 mm; the internal diameter was 20 mm. The electric field intensity was ca. 400 V cm^−1^, the operating temperature of the IMS cell was around 50 °C, while the pressure inside the cell was atmospheric pressure (ca. 1000 mbar). The radioactive ionization source was using the β-emitting isotope ^3^H (tritium), which was embedded within a stainless-steel disc; the initial activity of the ionization source was 300 MBq. Drift gas purified dry air at a flowrate of 400 cm^3^ min^−1^ was used, which was recirculated in a closed-loop pneumatic circuit that contained a filter with a 10A molecular sieve. 

**Figure 1:**
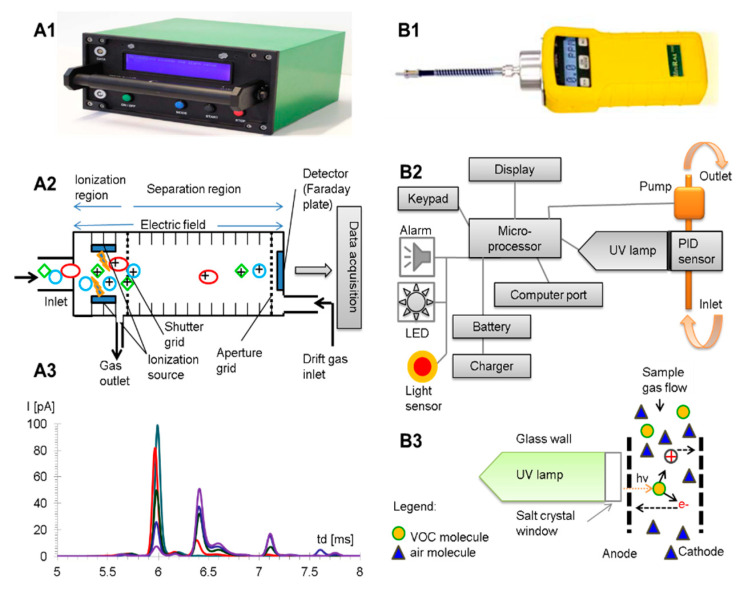
Schematic of the instrumentation used for the experiments. Part A: IMS, where (**A1**)—photo of IMS; (**A2**)—IMS cell and operating principle; (**A3**)—typical IMS response. Part B: PID ppbRAE Plus, where (**B1**)—photo of PID; (**B2**)—component parts of the PID system; (**B3**)—photoionization principle.

Sample air flow (50 cm^3^ min^−1^) was provided by a valve operated sequentially, and the inlet air flow (200 cm^3^ min^−1^) was secured by an inlet system using a sequentially pulsed valve. A minimal detectable concentration of vapors for an analyte is usually between 1 ppb_v_ and 100 ppb_v_ (depending on the proton affinities of the target analytes in the positive mode, or on the electron affinities in the negative mode, respectively). Power supply was given either by an internal rechargeable Li-Ion battery at 19 V DC (autonomous mode; min. operation time—ca. 8 h) or by mains (AC 220 V/50 Hz). The power consumption was only 6 W. The instrument was operated via a laptop computer, using the IMS Control Program Software, ver. 2.209 (IUT mbH). A photograph of the IMS instrument, together with a schematic of the IMS cell and a typical IMS response, are presented in Figure 1 (Part A).

The principle of IMS is based on separation of ions in the gaseous phase and at atmospheric pressure, based on the different drift velocities of the either positive or negative ions in a homogeneous DC electric field. The ionization process is a soft one, since the analyte molecules are not fragmented, and takes place in two stages—(a) formation of the reactant ions, followed by (b) generation of the product ions by collisional charge transfer from the reactant ions towards the analyte. Inside the radioactive ionization source, the first stage of ionization consists in formation of the so-called “reactant ions”, which are complex cluster ions like (H_2_O)_n_H^+^ (hydrated protons that form a vast majority), plus (H_2_O)_m_NH_4_^+^ and (H_2_O)_u_NO^+^ (in the positive ion mode) or (H_2_O)_n_O_2_^−^ (in the negative ion mode) [1,2]. Each time when the sample of air that contains the target analyte CS_2_ is being introduced inside the IMS system, the tritium-based radioactive ionization source transforms the neutral analyte molecules present in the sample in positive and/or negative product ions. In ion mobility spectrometry, the ionization process that occurs is a soft one, because the molecules of the analyte keep their identity, in opposition to electron-impact mass spectrometry, where they are fragmented. After these reactant and product ions are being formed inside the ionization region; they are periodically injected, by means of an electronic shutter called the “shutter grid”, inside the drift region (which is actually the space between the shutter grid and the ion collector). Ions are being propelled towards the detector (a Faraday plate) by a constant DC electric field E with a relatively low intensity, usually in the range 100–500 V cm^−1^. Ions will therefore travel inside the drift cell with a constant drift speed, v_d_, which has a value of ca. 5–10 m s^−1^. Of course, when a certain ionic species reaches the detector, a very low ionic current (typically below 100 nA) will be produced, and then amplified and displayed. Nevertheless, a certain chemical produces ions that have a characteristic drift speed in a neutral drift gas; in other words, a substance may be identified on the basis of its drift time t_d_ and consequently of its ion mobility K, which is nothing else than a constant linking the ion’s drift speed v_d_ to the intensity of the electric field: v_d_ = K × E = l_d_/t_d_ (l_d_ is drift length, and t_d_ is the drift time of a specific ion) [51]. Ion mobility can be therefore be expressed as K = v_d_/E = l_d_/(E⋅t_d_), while the reduced ion mobility K_0_ takes into account the effects of temperature and pressure: K_0_ = K⋅(T_ambient_/T_cell_)⋅(P_cell_/P_atmospheric_). Currently, reduced ion mobility K_0_ is used extensively as a qualitative parameter, in order to characterize a certain compound [1,2]. 

### 2.2. PID Instrument Used

A PID ppbRAE Plus Model PGM-7240, produced by RAE Systems Inc., Sunnyvale, CA, USA, was used for measuring the concentrations of CS_2_. This is a very sensitive photo-ionization detector (1 ppb_v_ to 200,000 ppb_v_), with a time response < 5 s, and which can be operated between −10 and +40 °C, used for measuring volatile photoionizable compounds. The PID instrument ppbRAE Plus has a highly compact design (size: 21.8 × 7.6 × 5.0 cm) with a mass of only 553 g and is equipped with a standard 10.6 eV UV lamp. The built-in sampling pump has an internal flow of 400 cm^3^ min^−1^. The PID instrument is powered by 4 AA alkaline batteries, which provide power for a duration of about 12 h of continuous operation. 

The data is initially stored by an internal microprocessor and can be subsequently transferred to the PC computer by using a serial COM connection cable and the dedicated software ProRAE Suite, ver. 3.01a, 2004. Prior to analyses, the instrument was calibrated with a calibration gas cylinder, purchased from RAE Systems, Inc., containing isobutylene with a concentration of 10 ppm_v_ in pure air. A PID photo, schematic of the component parts, and photoionization principle are presented in Figure 1 (Part B). 

### 2.3. Sampling and Work Flow Procedure

**Figure 2:**
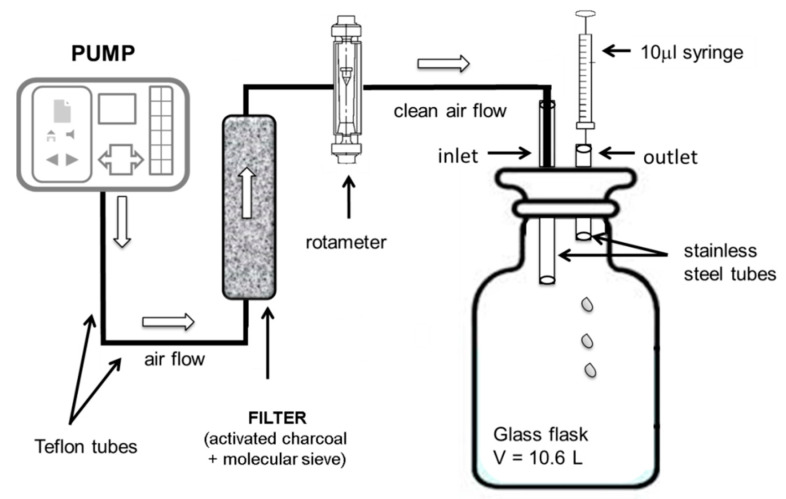
Schematic of the dynamic system used for the sampling.

CS_2_ (with ≥99.5% purity) was purchased from Sigma-Aldrich (St. Louis, MO, USA). Test standard atmospheres at trace levels (ppm_v_) with known concentrations were obtained by using the exponential dilution method. In order to obtain the mentioned test atmospheres, 10 μL of CS_2_ (the equivalent of 378 ppm_v_) was injected inside a glass flask having a volume of 10.6 L, through a lid equipped with two ports (the inlet and outlet). After injecting the liquid pure CS_2_, clean air was pumped inside the glass flask through the inlet port, while the outlet port was used for sampling air with the desired CS_2_ vapor concentrations. A schematic diagram of the dynamic system used for the sampling is presented in Figure 2.

The air flow provided by the pump was 1 L min^−1^, while the sampling procedure occurred during 90 min. After each minute, the gas flow was stopped and the remaining CS_2_ vapor concentration in the flask was measured by the ppbRAE PID. The IMS measurements started only when the CS_2_ concentration was lower than the level of ca. 15 ppm_v_, in order to avoid the IMS contamination and the subsequent unwanted memory effects due to heavy saturation of the IMS instrument with the analyte.

## 3. Results

**Figure 3:**
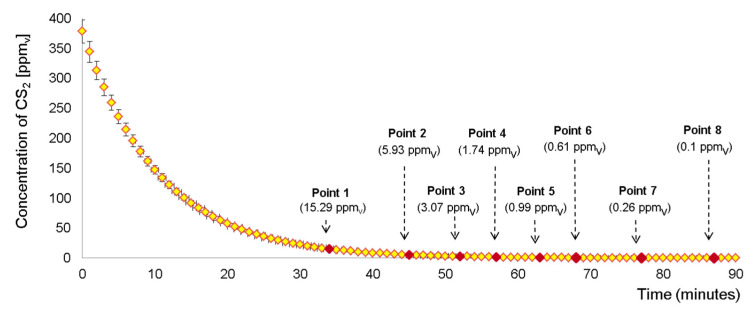
Evolution of the CS_2_ exponential dilution gathered with PID, as well as the sampling points used for the IMS measurements.

Experimental data generated by PID were continuously recorded during 90 min (one measurement per minute). Therefore, the PID readings were indicating the real concentration of the CS_2_ vapors inside the glass flask, at a certain moment in time. In the case of IMS, the measurements started when the vapor concentration arrived at 15 ppm_v_, and they were gathered simultaneously with the data produced by the PID instrument; eight different concentrations were investigated. The experiments were run in triplicate, during three different days, and a standard deviation between 7% and 2% was obtained for each concentration of CS_2_. The evolution of the analyte concentration during the exponential dilution experiment, obtained by using the PID device, is presented in Figure 3, in which the sampling points for the IMS measurements are marked as well. 

The ion mobility spectrometric response from CS_2_ was obtained in the negative ion mode, which constitutes a real advantage for the identification of this TIC—since fewer compounds produce IMS responses in the negative ion mode compared to those generating responses in the positive ion mode.

Table 3 summarizes, in a succinct, logical, and clear manner, the experimental results coming from both analytical techniques based on the ionization of the chemicals at atmospheric pressure—IMS and PID. The measured CS_2_ concentration using the PID instrument was calculated by multiplying the PID reading (in ppb_v_ units of isobutylene) with the PID correction factor for carbon disulfide (CF_CS2_ = 1.2) (for the UV lamp with a photon energy of 10.6 eV). We also have to emphasize at this point that the concentration of carbon disulfide vapors was checked continuously using the PID instrument ppbRAE Plus. In other words, by injecting a small volume of pure liquid CS_2_ (10 μL), we just intended to create a high initial concentration of CS_2_ vapors of ca. 380 ppm_v_, which has been further diluted using clean air in order to get a set of standard atmospheres with low vapor concentrations of carbon disulfide.

The ion mobility spectrometric response consisted of simple spectra, where one PIP (product ion peak) can be assigned to CS_2_ in the negative ion mode, at a drift time (t_d_) = 5.38 ms. The negative reactant ion peak (RIP) was noticed at a drift time (t_d_) = 5.68 ms. 

**Figure 4:**
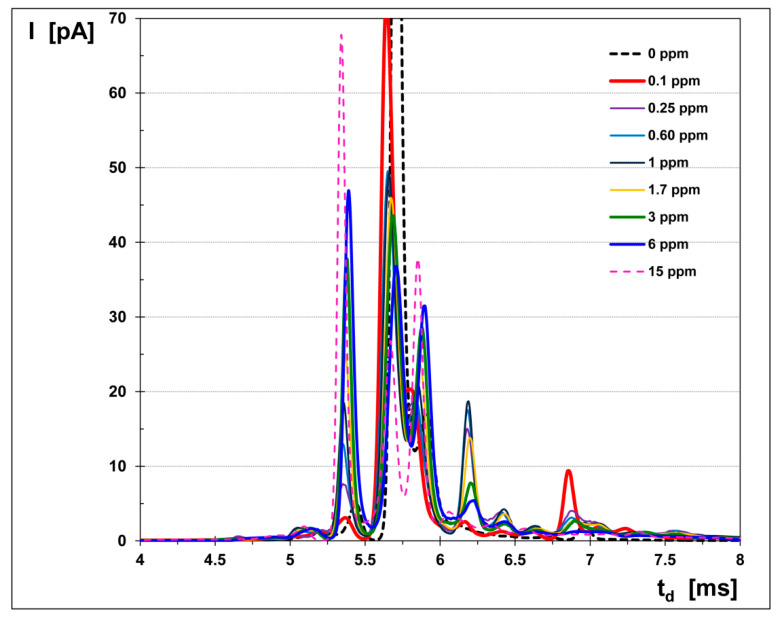
The ion mobility spectrometric response for CS_2_, obtained in the negative mode. Note: Although all spectra were collected from 1 to 20 ms, for the sake of clarity only the useful part of these spectra is presented—namely, the temporal interval, from 4 to 8 ms, which included all peaks.

The IMS spectra obtained in the negative mode are presented in Figure 4 and are perfectly illustrating the balance of the peaks’ intensity with the increase in the measured CS_2_ concentration. Thus, when the CS_2_ vapor concentration increases, the intensity of the negative RIP decreases while the height of the PIP increases. The peak observed at a drift time of ca. 6.2 ms is most probably generated by ultra-traces of acetic acid vapors, present as an impurity, since it remained relatively constant when the CS_2_ concentration changed.

The exact identity of both the reactant and product ions produced inside the IMS cell could be investigated and then assigned with a high degree of certainty only by coupling the IMS instrument with a mass spectrometer; this results in using a complex, hyphenated analytical system of the IMS-MS type. In fact, IMS-MS devices were used especially with the purpose of investigating the identity of the ions generated by highly toxic chemicals, such as chlorine and phosgene [9,10]. Identification of the ions generated by CS_2_ inside the IMS cell was not feasible in our study, because we have utilized only an IMS instrument. However, we can make the reasonable assumption that, in the negative ion mode, the product ion generated by carbon disulfide may be regarded as a sulfide negative ion.

**Figure 5:**
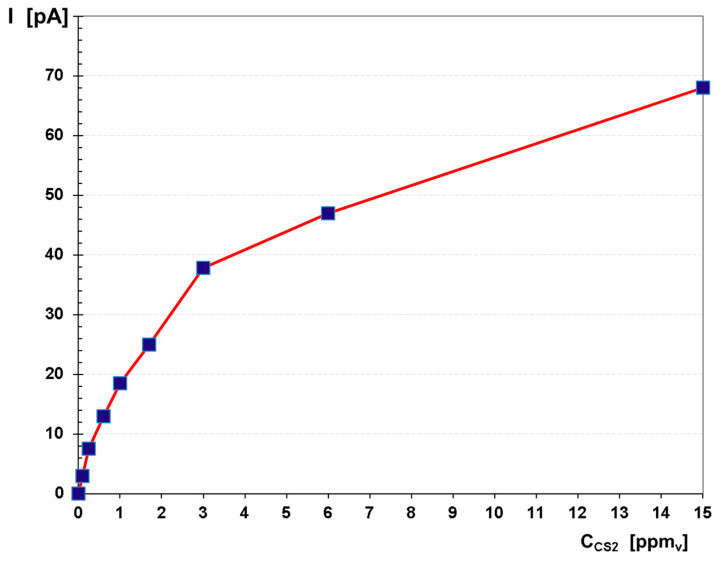
Calibration graphs for CS_2_ in the negative ion mode, normalized for the background air; the peak height of the single-product ion peak is plotted.

Quantitative data was used in order to build the calibration curve and then to evaluate the quantitative response of the ion mobility spectrometer to CS_2_. This calibration graph is given in Figure 5. A careful examination of both Figure 5 and of all the ion mobility spectra afferent to all the CS_2_ concentration levels (Figure 4) gives us the opportunity to extract the following conclusions:

1. Total disappearance of the reactant ion peak RIP did not happen. It means, therefore, that saturation of the IMS instrument was not reached. In other words, contamination of the IMS cell has been successfully avoided, and, consequently, we may rely on the quantitative data provided by the IMS device.
2. The minimum concentration measured was 100 ppb_v_ (ca. 0.31 mg m^−3^) and the estimated minimum concentration level is thought to be ca. 30 ppb_v_ (ca. 0.10 mg m^−3^).
3. Saturation is thought to occur at around 20 ppm_v_ (ca. 63 mg m^−3^) CS_2_, which is in total accordance with the well-known fact that the dynamic range of any IMS instrument that is equipped with a radioactive source extends to about two orders of magnitude.

The logarithmic-type calibration is totally specific to the IMS response from an instrument that uses a radioactive ionization source [1,2]. The negative ion mobility spectra obtained for the high concentrations (>3 ppm_v_) of analyte show a continuous decrease in the RIP amplitude with an increasing CS_2_ vapor concentration. However, even the spectra obtained for the maximum CS_2_ level, at 15 ppm_v_ (47.5 mg m^−3^), clearly showed that the saturation threshold has still not been reached, since the reactant ion peak (RIP) still keeps an intensity of ca. 20% of its value when the carbon disulfide concentration was zero. Thus, we can claim that the golden rule of avoiding a heavy contamination of the IMS cell and gas lines inside the IMS instrument has been successfully obeyed. A heavy contamination of any IMS system must be avoided at any price, because it will produce unwanted memory effects and subsequent false alarms as well.

Nevertheless, all IMS spectra provide highly valuable information—both qualitative information, useful for characterizing a certain chemical (which resides in the drift time (t_d_) and in the afferent reduced ion mobility (K_0_)), and quantitative information (included in the peak area/height). For any ionic cluster, the drift time is inversely proportional to mass and size, and proportional to its electrical charge. In summary, any peak of an ion mobility spectrum is characterized by a set of three numbers: (a) its drift time, t_d_ (in milliseconds); (b) its reduced ion mobility, K_0_ (in cm^2^ V^−1^ cm^−1^), and (3) its height (amplitude), h_max_ (in pA).

Qualitative information, which comprises the reduced ion mobility for both the negative reactant ion peak and for the CS_2_ product ion peak, is summarized in Table 4. A special mention to the fact that CS_2_ produces an ion that has a higher ion mobility (so travels faster) than the negative reactant ions; this situation is pretty rare and has been observed for very few target analytes, such as chlorine or hydrogen cyanide.

It is well worth mentioning here the ratio between the drift times of PIP and RIP, which is equivalent to the ratio of the reduced mobilities: t_d PIP_/t_d RIP_ = K_0 RIP_/K_0 PIP_ = 0.947.

For any analytical technique, including of course the IMS, the possible interferences are always a source of concern, for a variety of reasons—ranging from a loss in sensitivity to apparition of false alarms. However, it is absolutely needed to point out that in IMS only about 20% of the chemicals are generating product ions in the negative mode. Therefore, if a negative ion response is elicited, an ionization-based selectivity is obtained. In other words, the probability of having possible interfering chemicals in the negative operation mode is much lower, compared to the positive operation mode. 

Resolution of the Mini IMS instrument, which is defined as the ratio between the drift time of a certain ion and its width at half height (R_IMS_ = t_d_/Δt_d_), also has been calculated for both the negative RIP ion and PIP ion generated by CS_2_. The results are given in Table 5.

The resolution is very close to the value of 50 indicated by the manufacturer and has an excellent value compared to other commercial portable IMS instruments with miniaturized measure cells (with lengths of ca. 4–5 cm), which display a lower resolution, usually between 10 and 15.

## 4. Discussion

In conclusion, we can claim that the carbon disulfide vapors in air were successfully measured at the trace and ultra-trace levels, between 100 ppb_v_ and 15,000 ppb_v_, using IMS in the negative ion mode. Detection of CS_2_ in real time (several seconds) was successfully accomplished by IMS and by PID. Using IMS, a negative response was noticed, with one distinct and clearly separated product ion, with a reduced ion mobility, K_0_, of 2.25 cm^2^ V^−1^ s^−1^. In other words, the negative ion mode of the IMS response offers a good ionization-based selectivity in detection of CS_2_.

Quantitatively speaking, we emphasize that the concentration range that was investigated in the negative ion mode—from 0.1 to 15 ppm_v_ CS_2_—has provided highly relevant pieces of information related to both the measurement range and the saturation. We have noticed that saturation starts to appear after ca. 15 ppm_v_ of CS_2_. A minimum detectable concentration (MDC) of ca. 30 ppb_v_ (0.10 mg m^−3^) CS_2_ was also estimated. We were able to measure 100 ppb_v_ (0.31 mg m^−3^) of CS_2_ in air, a concentration that is 10 times lower than the REL TWA value (1 ppm_v_) and 5000 times lower than the IDLH value (500 ppm_v_). 

We conclude that the portable ion mobility spectrometer model Mini-IMS manufactured by IUT mbH Berlin has a very good sensitivity for CS_2_ vapors present in air samples and can therefore be very useful in real-life situations that necessitate rapid detection of carbon disulfide vapors. The IMS instrument must of course be evaluated in real-life scenarios too, and this field evaluation could be the object of future studies. In conclusion, portable IMS instrumentation proves to be an invaluable tool in quickly detecting traces of CS_2_ as a toxic industrial chemical and in protecting the health of workers.

### Validation

The suitability of the developed analytical method to its purpose was evaluated by using a simplified validation process, in which a set of parameters were assessed—limit of detection, limit of quantitation, linear range, sensitivity, accuracy, and trueness.

Limit of detection (LOD) is defined as being the lowest concentration that produces a signal-to-noise (S/N) ratio equal to 3, while limit of quantitation (LOQ) is defined as the lowest concentration that generates an S/N ratio equal to 10. The background signal—which is defined as the standard deviation of the background noise—was obtained by using the first 400 data points (IMS ionic currents for the time interval from 1.00 to 5.00 milliseconds, in increments of 0.01 millisecond) for every IMS spectrum; the resulting average value was s = 0.124 pA. Sensitivity (S) is defined as the increase in signal Y (in this case, peak height) with augmentation in concentration (S = ΔY/ΔC). Table 6 presents all these relevant figures of merit related to CS_2_ detection.

Precision was assessed by doing the analyses in triplicate (see Table 4). Accuracy was estimated by using the relative standard deviation (RSD), which was between 3.5% and 6.7% for the product ion peak generated by carbon disulfide, in the negative ion mode. A good repeatability of the results was noticed, since the RSD < 10%.

Traceability to a certified reference material (CRM), based on the standard of 10 ppm_v_ i-butylene in air, used for calibrating the PID instrument, was realized. We re-emphasize again that the PID instrument has been used to verify all CS_2_ concentrations when those atmospheres were simultaneously interrogated using the IMS instrument. Consequently, the use of this CRM is supporting the trueness of the experimental results.

Future research directions that may be approached are investigations of CS_2_ using different types of ion mobility spectrometers, such as aspiration IMS instruments (a-IMS) or differential mobility spectrometers (DMS).

## 5. Conclusions

The successful detection and quantification of toxic industrial CS_2_, using time-of-flight IMS, are described here for the first time. Carbon disulfide was determined in the negative ion mode. Simple spectra with just one product ion peak were obtained. Reduced ion mobility for CS_2_ was found to be K_0_ = 2.25 cm^2^ V^−1^ s^−1^. Measurement of CS_2_ at 0.1 ppm_v_ was realized, and an LOD of ca. 0.03 ppm_v_ was estimated. Saturation is thought to appear at concentration levels above 20 ppm_v_ CS_2_.

## Figures and Tables

**Table 1:** Formula, chemical, and physical properties of carbon disulfide (adapted from [16,17,18]).

Substance Name and Formula	Properties	Observations
Carbon disulfide CS_2_ (S=C=S) CAS#: 75-15-0 EC#: 200-843-6	Molecular mass: 76.15 g mol^−1^ Boiling point: 46.2 °C Melting point: −111.61 °C Density: 1.263 g cm^−3^ @ 20 °C Refractive index: 1.6319 @ 20 °C Flash point: −30 °C Auto-ignition temperature: 90 °C Relative density of vapors: 2.67 (air = 1) Vapor pressure: 360 mm Hg @ 25 °C Relative evaporation rate: 22.6 (Butyl acetate = 1) Ionization energy: 10.08 eV Vaporization enthalpy: 84.1 cal g^−1^ Octanol-water coefficient (log K_ow_): 1.94 Solubility: water solubility: 2.1 g L^−1^; Soluble in: ethanol, benzene, ether, chloroform Explosive limits: 1% vol. LEL; 50% vol. UEL	**Risks**: flammable in both liquid and vapors forms; causes serious skin and eyes irritation; affects fertility and is a teratogenic agent in case of repeated or prolonged exposure. **Reactivity**: very flammable; stable in storage; upon heating it decomposes to form toxic sulfur oxides; may explode on heating, shock or friction. **Conversion**: 1 ppm_v_ = 3.16 mg m^−3^ (20 °C)

**Table 2:** Emitted concentrations of CS_2_ as a result of rayon fiber production.

Country	Point of Measurement in Viscose Factory	Recorded Concentration (mg m^−3^)		Reference
		8-h TWA	Range	
Finland	viscose rayon fiber factory	9.4	4.7–25	[39]
	viscose sheeting production	13	0.6–28	
Yugoslavia	in the spinning rooms	63		[40]
	manufacturing departments	19		
Taiwan	in the cutting areas	125–210	470–940	[41]
	in the spinning areas		47–310	
	in the ripening area	170		[42]
	filament spinning	61		
Poland	synthetic fibers factory		9.4–23	[43]
Singapore	in a rayon factory		8.4–63	[44]
Germany	in viscose rayon factory		0.6–210	[45]
Belgium	centrifuge operator		3.1	[46]
	in the spinning areas		150	
Bulgaria	viscose rayon production facility		9.4–63	[47]
Canada	chemical company		310–630	[48]
Czechoslovakia	viscose rayon factories		30.4–152	[22]

**Table 3:** Summary of the quantitative results obtained from both the PID detector and the IMS instrument in the negative ion mode, respectively (three replicates were used for peak height, in order to calculate the standard deviation).

C_CS2_—Measured with PID	IMS Data—Negative Ion Mode	
	Drift Time t_d_ (ms)	Peak Height h_max_ (pA)
0 ppb_v_	NEG RIP 5.68	98.0 ± 4.2
0.10 ppm_v_	PIP 5.38	3.0 ± 0.2
0.25 ppm_v_	PIP 5.38	7.5 ± 0.4
0.60 ppm_v_	PIP 5.38	13.0 ± 0.8
1.00 ppm_v_	PIP 5.38	18.5 ± 1.1
1.70 ppm_v_	PIP 5.38	25.0 ± 1.3
3.00 ppm_v_	PIP 5.38	37.8 ± 1.6
6.00 ppm_v_	PIP 5.38	47.0 ± 1.9
15.00 ppm_v_	PIP 5.38	68.0 ± 2.4

**Table 4:** Reduced ionic mobilities, K_0_, calculated for both the reactant ions and for the ions produced by CS_2_.

Operation Mode	Ion Drift Time, t_d_ (ms)	Drift Speed, v_d_ = l_d_/t_d_ (m s^−1^)	Ion Mobility, K (cm^2^ V^−1^ s^−1^)	Reduced Ion Mobility ^1^, K_0_ (cm^2^ V^−1^ s^−1^)
Negative	RIP: 5.68	9.68	2.421	2.130
	PIP: 5.38	10.22	2.556	2.249

^1^—Experimental conditions were l_d_ = 5.5 cm; E = ca. 400 V cm^−1^; P = 1003 mbar; T = 50 °C. Therefore: K = v_d_/E and K_0_ = (1/t_d_) × (5.5⋅1003⋅293.15⋅10^3^)/(400⋅1013.25⋅323.15) = 12.10/t_d_.

**Table 5:** Resolution of the Mini IMS for CS_2_.

Ion Drift Time, t_d_ (ms)	Peak Width Al Half Maximum, Δt_d_ (ms)	Resolution, R_IMS_
RIP: 5.68	0.13	43.7
PIP: 5.38	0.13	41.4

**Table 6:** Figures of merit related to the IMS detection of CS_2_ in the negative ion mode.

Ion Mode	LOD (ppb_v_)	LOQ (ppb_v_)	Linear Range (ppb_v_)	Equation	R^2^	S (pA/ppm_v_)
Negative	27	90	90–3000	Y = 11.462⋅X + 4.763	0.978	12.6

## Supplementary Materials

The following are available online at https://www.mdpi.com/2305-6304/8/4/121/s1, Table S1: The effect of prolonged exposure at CS_2_ in humans.
