# Supplementary material for: Hunting for Toxic Industrial Chemicals: Real-Time Detection of Carbon Disulfide Traces by Means of Ion Mobility Spectrometry
Source: Toxics. 2020 Dec 14;8(4):121. doi: 10.3390/toxics8040121 (PMC7765148; doi:10.3390/toxics8040121)
Supplement: Supplementary file 1 [file toxics-08-00121-s001.pdf]

Supplementary Materials: Hunting for Toxic Industrial Chemicals: Real time detection of carbon disulfide traces by means of Ion Mobility Spectrometry

Victor Bocos-Bintintan and Ileana Andreea Ratiu

Table S1. The effect of prolonged exposure at CS_2 in humans (adapted information [1]).

Affected System/ Effects	Time Exposure @ Given Level Of Cs_2 (ppm)	Exposed Subjects	Observed Effects
Neurological	Accidentally exposure to high concentrations	123	Neurophysiological and behavioral effects, patho-morphology of peripheral nervous system structures; fainting and loss of consciousness.
	15 years (l0–20)	118	Reduced maximal motor conduction velocity of the median, ulnar, peroneal, and posterior tibial nerves.
	2 years (150–300)	19	Polyneuritis and overt polyneuropathy.
	Long-term exposure (it is supposed that safe levels have been exceeded)	21	Distal sensory shading indicated by decreased sensitivity to pinprick and light touch, intention tremors, resting tremors, and nerve conduction abnormalities, Parkinson.
	Long-term exposure (19.3–57.8)	17	Anxiety, introversion, depression, deficient attention, speediness, and carefulness.
Gastrointestinal	4.2 years (l–36)	123	Nausea, vomiting, impaired appetite.
Hematological	2–8 years (59–169)	57	Decrease of serum plasmin concentrations.
Hepatic	4.2 years (l–36)	119	Significantly increased liver size and γ-glutamyl-transferase activity.
Renal	5 years (0–21)	114	Slight increase in plasma creatinine concentration.
Endocrine	20 years (0.008–0.02)	50	Depression in serum thyroxine.
Ocular Effects	17 years (15–30)	n/a	Increased frequency of micro-aneurysms, dot hemorrhages or micro-aneurysms of retina, inflammatory degenerative changes in retinas.
Body Weight	4.2 years (l–36)	119	Anorexia.
Other Systemic Effects	< 5 years (4.8–8)	n/a	Significantly elevated plasma sodium & chloride ions; decreased erythrocyte potassium & calcium.
Reproductive Effects	Years (12–18)	n/a	*Women:* menstrual disorders, toxemia of pregnancy.
	10–36 years (<10)	15	*Men:* significantly increased serum follicle-stimulating hormone & luteinizing hormone; primary gonadal insufficiency.

Reference

1. *U.S. Department Of Health And Human Service Public Health Service Agency for Toxic Substances and Disease Registry. Toxicological Profile for Carbon Disulfide*; ATSDR. Atlanta, GA, USA, 1996.

© 2020 by the authors. Licensee MDPI, Basel, Switzerland. This article is an open access article distributed under the terms and conditions of the Creative Commons Attribution (CC BY) license (http://creativecommons.org/licenses/by/4.0/).
